# An extracellular matrix-mimetic coating with dual bionics for cardiovascular stents

**DOI:** 10.1093/rb/rbad055

**Published:** 2023-05-30

**Authors:** Nuoya Chen, Mingyu Li, Haoshaung Wu, Yumei Qin, Jian Wang, Kai Xu, Rifang Luo, Li Yang, Yunbing Wang, Xingdong Zhang

**Affiliations:** National Engineering Research Center for Biomaterials, Sichuan University, Chengdu 610064, China; National Engineering Research Center for Biomaterials, Sichuan University, Chengdu 610064, China; National Engineering Research Center for Biomaterials, Sichuan University, Chengdu 610064, China; National Engineering Research Center for Biomaterials, Sichuan University, Chengdu 610064, China; Shanxi Key Laboratory of Functional Proteins, Shanxi Jinbo Bio-Pharmaceutical Co., Ltd, Taiyuan 030032, Shanxi, China; Department of Cardiology, General Hospital of Northern Theater Command, Shenyang 110000, China; National Engineering Research Center for Biomaterials, Sichuan University, Chengdu 610064, China; National Engineering Research Center for Biomaterials, Sichuan University, Chengdu 610064, China; National Engineering Research Center for Biomaterials, Sichuan University, Chengdu 610064, China; National Engineering Research Center for Biomaterials, Sichuan University, Chengdu 610064, China

**Keywords:** cardiovascular stent, recombinant humanized collagen type III, extracellular matrix, anti-coagulation, endothelialization

## Abstract

Anti-inflammation and anti-coagulation are the primary requirements for cardiovascular stents and also the widely accepted trajectory for multi-functional modification. In this work, we proposed an extracellular matrix (ECM)-mimetic coating for cardiovascular stents with the amplified functionalization of recombinant humanized collagen type III (rhCOL III), where the biomimetics were driven by structure mimicry and component/function mimicry. Briefly, the structure-mimic was constructed by the formation of a nanofiber (NF) structure via the polymerization of polysiloxane with a further introduction of amine groups as the nanofibrous layer. The fiber network could function as a three-dimensional reservoir to support the amplified immobilization of rhCoL III. The rhCOL III was tailored for anti-coagulant, anti-inflammatory and endothelialization promotion properties, which endows the ECM-mimetic coating with desired surface functionalities. Stent implantation in the abdominal aorta of rabbits was conducted to validate the *in vivo* re-endothelialization of the ECM-mimetic coating. The mild inflammatory responses, anti-thrombotic property, promotion of endothelialization and suppression of excessive neointimal hyperplasia confirmed that the ECM-mimetic coating provided a promising approach for the modification of vascular implants.

## Graphical Abstract

**Figure:**
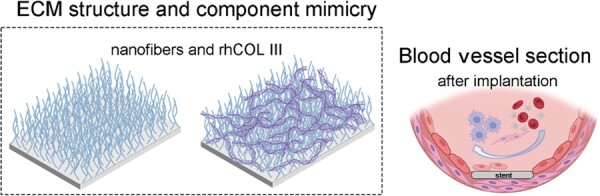

## Introduction

Percutaneous coronary intervention (PCI) with a vascular stent is the most successful treatment for coronary heart diseases [1, 2]. Although the risk of in-stent restenosis (ISR) has decreased after the advent of drug-eluting stent (DES) [3, 4], anti-proliferative drugs on the stent [such as paclitaxel (PTX) and sirolimus] for suppressing the proliferation of smooth muscle cell (SMC) also inhibit the endothelialization process after stent implantation. The delayed endothelialization caused by these drugs poses a risk for the incomplete healing of neointima and thus leading to late thrombosis (LST), threatening the life of patients [5, 6]. Currently, bioresorbable vascular stents (BRS) are designed to eliminate foreign body reactions (FBR) that arise from non-degradable metallic stents [7], since surrounding tissues absorb it completely and leave only healthy vessels [8, 9]. However, currently applied coatings on BRS in the clinic follows the strategy of the coatings on non-degradable metallic DES, which means LST may also occur on BRS. Therefore, it is essential that desired BRS coating should induce a mild inflammatory response, suppress the excessive hyperplasia of SMCs and promote re-endothelialization [10].

Resting endothelial cells (ECs) are known to inhibit coagulation, but during PCI surgery, injury and stimulation can contribute to a decrease in anti-coagulation and leukocyte recruitment [9]. In addition, rapid inflammatory responses occur, accompanied by monocyte aggregation around the stent and their differentiation into macrophages [10, 11]. The damage to endothelial layer during PCI is unavoidable, as both acute inflammatory responses and thrombus deposition can lead to delayed endothelialization and excessive hyperplasia, eventually resulting in the failure of stent implantation [11, 12]. Since many complicated biological responses around the material/vessel interface have a vital impact on the efficiency of implantation, multi-functional modification, including anti-inflammation, anti-coagulation, and rapid endothelialization functions, is a promising trajectory for the design of an ideal coating for vascular stent [13–16]. Many researchers have constructed a multi-functional surface via multiple substances. Yang *et al.* reported a phenolic-amine chemistry-mediated coating, which suppressed the inflammation via tannic acid (TA) and enhanced the anti-coagulation ability via thrombin inhibitor bivalirudin (BVLD) [17]. In addition, the vascular endothelial growth factor (VEGF) is widely applied to vascular stent modification. Sun *et al.* designed a stent coated with bi-layered PLGA nanoparticles containing VEGF and PTX [18]. PTX suppressed SMC proliferation, and VEGF promoted rapid re-endothelialization. Biomaterials, such as TA, hyaluronic acid (HA), and epigallocatechin gallate, have been widely applied in the modification of cardiovascular stents. These materials are often combined with other substances (i.e. heparin, BVLD and PTX) to create stent coatings [19–21]. The application of most multi-functional coatings is limited by the high price and complicated assembly process. In addition, the introduction of multiple biomaterials may impact the functions of these substances. Hence, introducing a single biomacromolecule to meet the multi-functional requirements of stents could be a novel and promising method for modifying cardiovascular materials.

Recombinant humanized collagen is a designable, water-soluble and biocompatible material [22–24]. However, its application for blood-contacting modification is impeded by the activation and adhesion of platelets. Considering this, we designed a tailored recombinant protein containing cell adhesive motives, Gly–Glu–Arg (GER) and Gly–Glu–Lys (GEK) triplets [25], referred to as recombinant humanized collagen type III (rhCol III). Compared to most recombinant collagens, rhCol III shows great anti-coagulation properties, due to the deletion of hydroxyproline-containing fragments, which is known to bind surface integrin α2β1 to activate platelets [26]. In addition, rhCol III also exhibits anti-inflammatory capacities and selectively promotes EC proliferation [22, 24, 26]. As previously reported, the layer-by-layer (LBL) coating of HA and rhCol III is a promising blood-contact material [26]. However, it is still far from ideal as a vascular stent coating due to the tedious assembly process of LBL, the lack of stability, and the limited introduction of rhCol III. Therefore, it is important to construct a stable coating using single rhCol III and explore the efficiency of its functions in response to complicated biological environments.

Mimicking the extracellular matrix (ECM) is a widely accepted approach for modifying scaffolds. The inherent fiber structure and functional matrix components (i.e. collagen, laminin and fibronectin) could help to support cell/material interactions [26–30]. To design an ECM-mimetic coating with both structure-mimic and function-mimetic could benefit from topographical surface and multiple functions of components. The polymerization of polysiloxane has been used to construct fibrous or porous network [31–33]. Furthermore, the polymerization of polysiloxane to form a nanofiber (NF) structure can enable the loading of functional components onto a three-dimensional reservoir and then the immobilization of more functional components. Based on this, we designed an ECM-mimetic coating with amplified functionalization of rhCOL III, where the biomimetics were driven by both structure mimicry (a fibrous network layer) and component/function mimicry (an rhCOL III-based matrix that supports cell growth and anti-coagulant ability).

**Figure 1.**
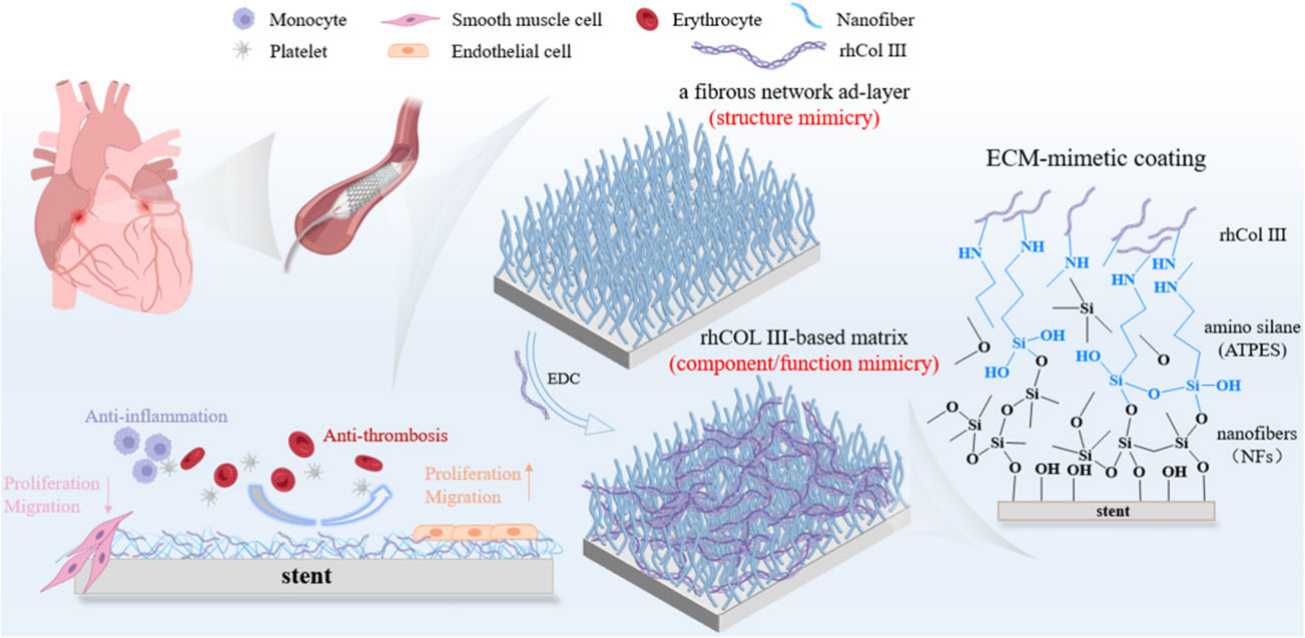
The component and multi-function of the rhCOL III (NF) coating. The surface of coronary stent was modified by nanofiber (NF), amino silane and rhCOL III, successively. The anti-thrombosis, anti-inflammation and enhanced EC proliferation functions of these ECM-mimetic coatings were studied.

As shown in Fig. 1, the ECM-mimetic coating was prepared in three steps, including basic NF coating formation, amino-functionalized substrate construction, and rhCol III covalent immobilization. It was designed to inhibit inflammation, reduce thrombosis, suppress intimal hyperplasia, and promote endothelialization. The NFs were formed on a Fenton-treated surface, where oxidative functional groups were introduced via the hydrolyzation and cross-linking of methyl trichlorosilane (MTS), followed by further introduction of amino groups by (3-aminopropyl) triethoxysilane (ATPES), which hydrolyzed and bound to the Fenton-treated NFs. The rhCol III was immobilized via the condensation reaction (carboxyl and amino groups) to construct the dual ECM-mimetic coating. Subsequently, the hemocompatibility and cytocompatibility were evaluated *in vitro*. Animal experiments were performed to test the anti-thrombosis and anti-inflammatory capacities, and *in vivo* vascular implantation was conducted to evaluate the potential of endothelialization and anti-hyperplasia. For further comparison, rhCol III was directly immobilized on the PLA substrate, to demonstrate the positive effect of the nanofibrous layer (NF layer) on rhCol III immobilization.

## Materials and methods

### Materials

Polylactic acid (PLA) sheets and vascular stents were provided by Sichuan Xingtai Pule Medical Technology Co., Ltd (Chengdu, China). The recombinant humanized collagen type III (rhCol III) was specially prepared and purified in Shanxi Jinbo Bio-Pharmaceutical Co., Ltd (Taiyuan, China). MTS and ATPES were purchased from Aladdin (Shanghai, China). 1-Ethyl-3-(3-dimethylaminopropyl) carbodiimide (EDC) and 2-(*N*-morpholino) ethane sulfonic acid (MES) were purchased from Adamas-beta (Shanghai, China). Iron (II) sulfate heptahydrate (FeSO_4_), hexane (≥98.0%), and ethanol were obtained from Chengdu Jinshan Chemical Reagent Co., Ltd (Chengdu, China). Fluorescein isothiocyanate (FITC) was obtained from Beijing Solarbio Science &Technology Co., Ltd. (Beijing, China).

### Fabrication of rhCol III coatings

The PLA sheets were successively cleaned with acetone, ethanol, and ultrapure (UP) water. Hydroxyl groups on the PLA substrate were introduced via Fenton’s reagent (pH 2.5–3.0), in which FeSO_4_ and hydrogen peroxide (H_2_O_2_) reacted with the substrate. The plates were immersed in hexane containing 0.2% vol MTS for 12 h, prior to being covered by NFs. Before coating of rhCol III, the PLA plates and stents were treated by Fenton reagent and then immersed in the ethanol of 2% vol ATPES for 2 h to form amino groups. The substrates were washed with ethanol and UP water, and immersed in the MES buffer solution (pH 5.0) containing EDC (0.08 M) and rhCol III (2 mg/ml). The sheets only covered by rhCol III were omitted in the preparation of NF.

### Characterization of rhCol III coatings

The elemental composition was analyzed by X-ray photoelectron spectroscopy (XPS, Thermo Fischer, ESCALAB Xi+, USA). The chemical bonds were evaluated by attenuated total reflectance-Fourier-transform infrared spectrometry (ATR-FTIR, Thermo Fisher Nicolet Is5, USA). The morphology of the samples and the diameter of NF were observed using a scanning electron microscope (SEM; FEI, USA) and atomic force microscopy (Bruker Dimension Icon AFM, USA). Water contact angles (WCA) were estimated using ThetaFlex (Biolin Scientific, Finland).

### Stability tests

The FITC-rhCol III was synthesized by mixing of rhCol III and FITC in a saturated sodium bicarbonate solution for 24 h. The solution was dialyzed in the dialysis tubing (3.5 kD) for 7 days and then freeze-dried. The preparation of FITC-rhCol III coatings was the same, but rhCol III was replaced with FITC-rhCol III. The samples were immersed in PBS on the shaker at 37°C and removed after 7, 14 and 28 days to observe the distribution of FITC-rhCol III under a laser-scanning confocal microscope (LSM880 Airyscan with STEDYCON, Carl Zeiss, Germany).

### Blood compatibility

#### Platelet adhesion test

Platelet-rich plasma (PRP) was collected from the supernatant of New Zealand white rabbit blood centrifugated at 1,500 r/min for 15 min. The PRP was incubated on the samples for 1 h at 37°C. Next, the samples were washed with PBS and immersed in 2.5% glutaraldehyde solution overnight for fixation [14, 34]. Then, the platelet morphologies were observed by SEM after dehydration by gradient alcohol [35].

#### *Ex vivo* blood circulation thrombogenicity test

The *ex vivo* blood circulation test was carried out on New Zealand white rabbits (2.5–3 kg), as the arteriovenous shunt model is a common approach to evaluate blood compatibility. The coatings of rhCOL III with and without NF were prepared on the polyvinyl chloride tubes. The tubes were connected from the carotid artery to the jugular vein to form a blood circuit for 2 h. Then, the blood clots and the cross-section of the tubes were photographed and recorded for further evaluation of thrombosis.

### *In vitro* human vascular cell compatibility test

#### EC compatibility test

Second-generation human umbilical vein endothelial cells (HUVECs) were cultured in Dulbecco’s modified Eagle’s medium (DMEM) containing 10% (v/v) fetal bovine serum (FBS) and 1% (v/v) penicillin–streptomycin at 37°C in an atmosphere with 5% carbon dioxide [36]. The 5 mm × 5 mm sheets were sterilized under ultraviolet radiation and cultured with 0.5 ml ECs suspension (2 × 10^4^ cells/ml) for 24 and 72 h. The cell viability at 24 and 72 h was measured by cell-counting kit-8 assay (DMEM containing 10% CCK-8). The average optical density was measured on a Synergy H1 Multi-Mode Microplate Reader (BioTek, USA) at 450 nm. The HUVECs’ cytoskeleton was labeled with TRITC Phalloisin (red), and the nucleus was stained with 4',6-diamidino-2-phenylindole (DAPI, blue). Then, the HUVECs’ morphology was observed under a laser-scanning confocal microscope [37].

#### Smooth muscle cells compatibility test

Primary human umbilical artery smooth muscle cells (HUASMCs) were grown in DMEM containing 10% (v/v) FBS and 1% (v/v) penicillin–streptomycin. The subsequent processes were similar to those mentioned above in the HUVEC compatibility test.

#### Macrophage cell compatibility test

RAW 264.7 mouse leukemia cells were cultured as the similar method mentioned in the HUVECs compatibility test, and the media after incubation were collected to determine the inflammatory factors. The expression of tumor necrosis factor-alpha (TNF-ɑ) and interleukin-10 (IL-10) in cell supernatant was measured by ELISA Kit (Jiangsu Meimian Industrial Co., Ltd, Yancheng, China).

### Subcutaneous implantation test

The animal experiments were carried out in accordance with the Animal Care and Use Committee of Sichuan University. Male adult Sprague-Dawley rats (Chengdu Dashuo Experimental Animal Co., Ltd, Chengdu, China) under anesthesia were implanted with 6 mm × 6 mm samples subcutaneously. The samples with surrounding tissue were removed and fixed in 4% paraformaldehyde for at least 24 h after 15 and 30 days. The surrounding tissue was stained with hematoxylin and eosin (H&E), as well as immunofluorescence (anti-CD68), to evaluate the thickness of the fibrous capsule and inflammation.

### Stent implantation evaluation

The rhCol III (NF) and bare stents were crimped before the experiment. Then, the sterilized stent was implanted in the abdominal aorta of the rabbit. After 1 and 3 months of implantation, the stents and surrounding vascular tissues were excised and washed with 800 U heparin solution to avoid clot deposition in the separate vessels. Then, the vessels containing stents were immersed in 4% paraformaldehyde for 1 week and stained with H&E and immunohistochemistry antibodies [anti-CD68, -CD31, -eNOS and -ɑ-smooth muscle actin (α-SMA)] for further evaluation.

### Statistical analysis

The stained slices were analyzed by Image-Pro Plus 6.0, and statistical analyses (more than three parallel samples) were conducted using GraphPad Prism 8.0, including one-way analysis of variance (ANOVA) and *t*-test to determine the statistical significance (*P < 0.05, **P < 0.01, ***P < 0.001 and ****P < 0.0001). All of the results are expressed as mean ± standard deviation (SD).

## Results and discussion

### Characterization of the NF substrates

**Figure 2.**
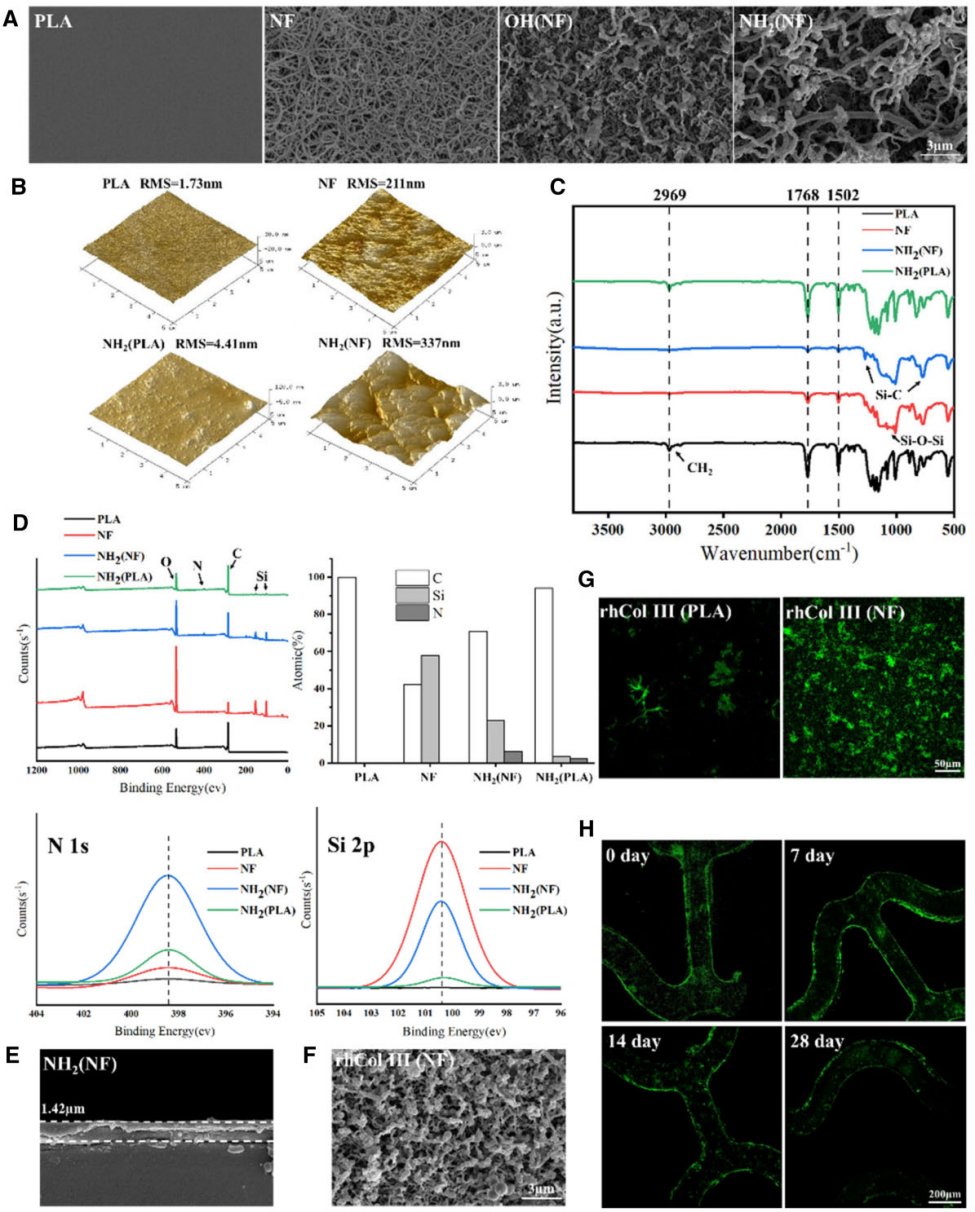
Characterization of coatings. (**A**) SEM and (**B**) AFM images of the PLA substrate and coatings. (**C**) Spectra of modified substrates tested by ATR-c. (**D**) XPS wide scan spectra of the surface elements with a narrow spectrum of N 1 s and Si 2p. (**E**) Cross-section and (**F**) morphology of NH_2_ (NF)-coated PLA observed by SEM. (**G**) Fluorescence signal on rhCOL III (PLA) and rhCOL III (NF). (**H**) Fluorescent microscopy images of rhCOL III (NF) after 7-, 14- and 28-day immersion in PBS.

The synthesis of NFs was conducted through the hydrolysis of MTS, which reacted with the hydroxy groups of the substrate treated with Fenton’s reagent and induced the polymerization of polysiloxane on the surface. As shown in Fig. 2A, the surface of PLA was smooth and flat. When the substrate was covered by NFs, the roughness increased from 1.73 to 211 nm (Fig. 2B), and the mean diameter of NF was about 119.4 nm. After undergoing the Fenton and ATPES processes, NFs were linked with hydroxyl groups and amino groups, and the mean diameters were 232.6 and 343.1 nm, respectively (Fig. 2A). The roughness was significantly different between amino-silanized bare PLA (RMS = 4.41 nm) and NF substrate (RMS = 337 nm) on account of the surface-to-volume ratio (Fig. 2B). This indicates that more NH_2_ groups could be immobilized on the PLA surface. The increase in the nitrogen (N) content in Fig. 2D also confirmed that more amino groups were connected to the NF substrate. In Fig. 2C, the FTIR spectra of the NF substrate had a new peak at 1070 cm^−1^, compared with the bare PLA, and this peak was assigned to the stretching vibration of Si-O-Si [32]. After APTES modification, the peak near 1270 cm^−1^ was enhanced due to the Si-C bond [33]. In addition, the peak near 1600 cm^−1^ was attributed to carbonyl stretching, and its intensity weakened because of the complete coverage of NFs. In contrast, the spectra of NH_2_ (PLA) were similar to the bare PLA because the coating thickness was not sufficient to measure, while the thickness of NH_2_ (NF) coatings was 1.42 µm according to Fig. 2E.

In summary, the fibrous network layer was successfully prepared on the PLA substrate to mimic ECM structure and provided a three-dimensional reservoir for more amino immobilization. In addition, the layer was hydrophobic because the WCA is about 162.6° (Supplementary Fig. S1). The NFs on the surface presented overhanging morphology and low surface energy. Thus, the water drops rested on the protrusions, entrapping air underneath and eventually rolling off the substrate [31, 32].

### Characterization of the rhCOL III coatings

FITC-rhCOL III and rhCOL III were immobilized on the amino-silanized NF substrates as mentioned above. The rhCOL III particles and NH_2_ groups were closely integrated via amide bonds, due to a chemical reaction between rhCOL III carboxyl and amino on the substrate (in MES buffer and EDC activation). Figure 2F presents the morphology after the immobilization of rhCOL III, and the conglutination of spherical particles was observed around NFs. In Fig. 2G, the fluorescence signals on FITC-rhCOL III (NF) were much stronger than that on FITC-rhCOL III (PLA) coatings, which confirmed that more rhCOL III was immobilized on the NF substrate. This phenomenon indicates that the fiber network interface increased the amount of rhCOL III immobilization.

The stability of the coatings has a significant impact on the effectiveness of implantation, and the function of the coatings should be maintained for at least 7 days, as a modified blood-contact device. As shown in Fig. 2H, the PLA stent coated with FITC-rhCOL III (NF) presented strong fluorescence signals before immersion. To test the coating stability, the FITC-rhCOL III (NF) stent was immersed in PBS solution, and the loss of rhCOL III after 7, 14, and 28 days was observed. There was no major attenuation in the fluorescence on the immersed stent after 7 days. After 14 days of immersion, a slight decrease in fluorescence indicated that the rhCOL III content of the coating decreased. Fluorescence signals were detected after 28 days of immersion, implying that the coating had good stability and could maintain long-term function after implantation.

The above results indicate that the introduction of a fibrous network layer increased the space for rhCOL III immobilization and the coating was stable due to the covalent binding between rhCOL III and NF layer. With the immobilization of hydrophilic rhCOL III, the rhCOL III (NF) coating was hydrophilic (Supplementary Fig. S1) on account of a 2D capillary effect [38]. The dual ECM-mimetic coating was a biomimetics driven by a fibrous network layer and a rhCOL III-based matrix. For further investigation of the advantages of dual bionics, the rhCOL III (PLA) coating (single ECM component mimetic) was prepared and evaluated.

### Anti-coagulant performance

**Figure 3.**
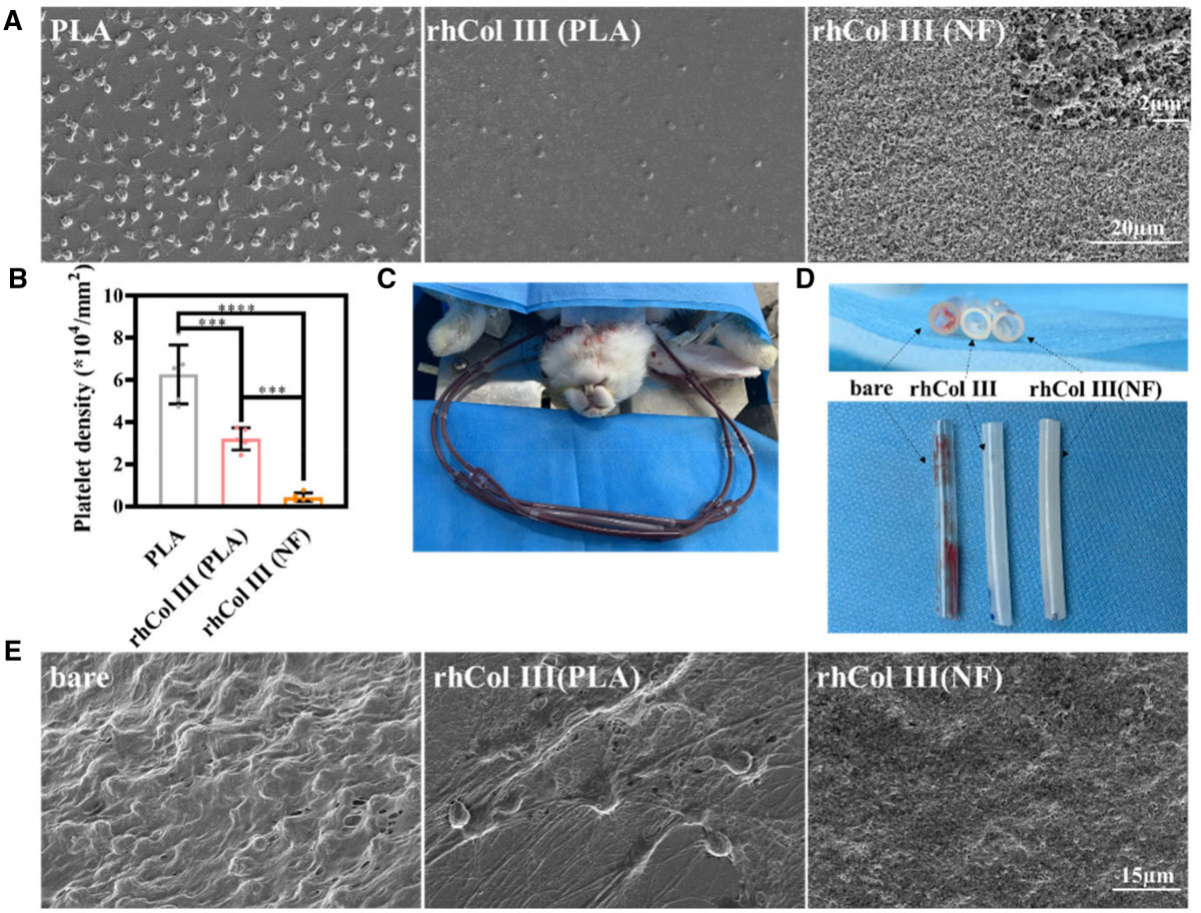
The hemocompatibility test. (**A**) Platelet adhesion/activation on the PLA, rhCOL III (PLA) and rhCOL III (NF), respectively. (**B**) The density of adhered platelets on the PLA, rhCOL III (PLA), and rhCOL III (NF) sheets. (**C**) The *ex vivo* arteriovenous shunt model. (**D**) Photo of the arteriovenous extracorporeal circuit and the deposited thrombus. (**E**) SEM images of the deposited components on the different surfaces after circulation for 1 h. *P < 0.05, **P < 0.01, ***P < 0.001, ****P < 0.0001 (one-way ANOVA, error bars are defined as SD).

Incubation with PRP to observe platelet adhesion and activation on the substrate is a common way to test the anti-coagulant performance *ex vivo*. As shown in Fig. 3A, a large number of activated platelets were adhered to the PLA sheet, as observed by SEM. In contrast, platelets were fewer and exhibited low activation on the rhCOL III (PLA) and rhCOL III (NF) substrates. After analyzing the density of platelets on the sheets in Fig. 3B, rhCOL III could obviously suppress platelet adhesion and activation and the anti-coagulation of rhCOL III (NF) coating was better. The rhCOL III (NF) substrate in Fig. 3A displays a larger image of the platelets on the rhCOL III (NF) coating, and the platelets observed were not active. A previous study demonstrated the efficiency of rhCOL III in repressing platelet adhesion [26], while the NF layer exhibited a micro/nanostructure and decreased the contact point for platelet adhesion [32]. In brief, the rhCOL III (NF) coating amplified the anti-coagulation of rhCOL III and influence platelet/material interaction, which had a positive effect on the resistance of platelet adhesion.

To further investigate the anti-thrombotic performance of the coatings, an *ex vivo* circulation from the carotid artery to the jugular vein of the rabbit was constructed to evaluate the samples in flowing blood. As shown in Fig. 3D, the bare tube was covered by severe thrombus deposition after 1 h of circulation, while there was little thrombus deposition on the rhCOL III (PLA) and rhCOL III (NF) samples from macroscopical observation. Moreover, the inner side of the tubes was observed by SEM, indicating the adhesion of fibrous protein and the activation of platelets. In Fig. 3E, the bare tube was filled with severe thrombosis, which consisted of a large amount of activated platelets and cross-linked fibrin. The increased fibrin network density promoted the aggregation of platelets and erythrocytes, which gave rise to the formation and deposition of thrombosis [39]. Due to the immobilization of rhCOL III, fewer platelets adhered to the rhCOL III (PLA) groups, compared with bare PLA. There were few platelets adhered on the rhCOL III (NF) surface. The coating construction in the tube was different from that on the PLA substrate, and the compact nanoparticles constituted the surface, instead of NFs. This was due to the hydrolyzation of the flowing MTS (Supplementary Fig. S2). The results of the PRP incubation and the *ex vivo* perfusion experiment proved the rhCOL III (NF) coating had good anti-thrombotic properties.

### Cell viability

**Figure 4.**
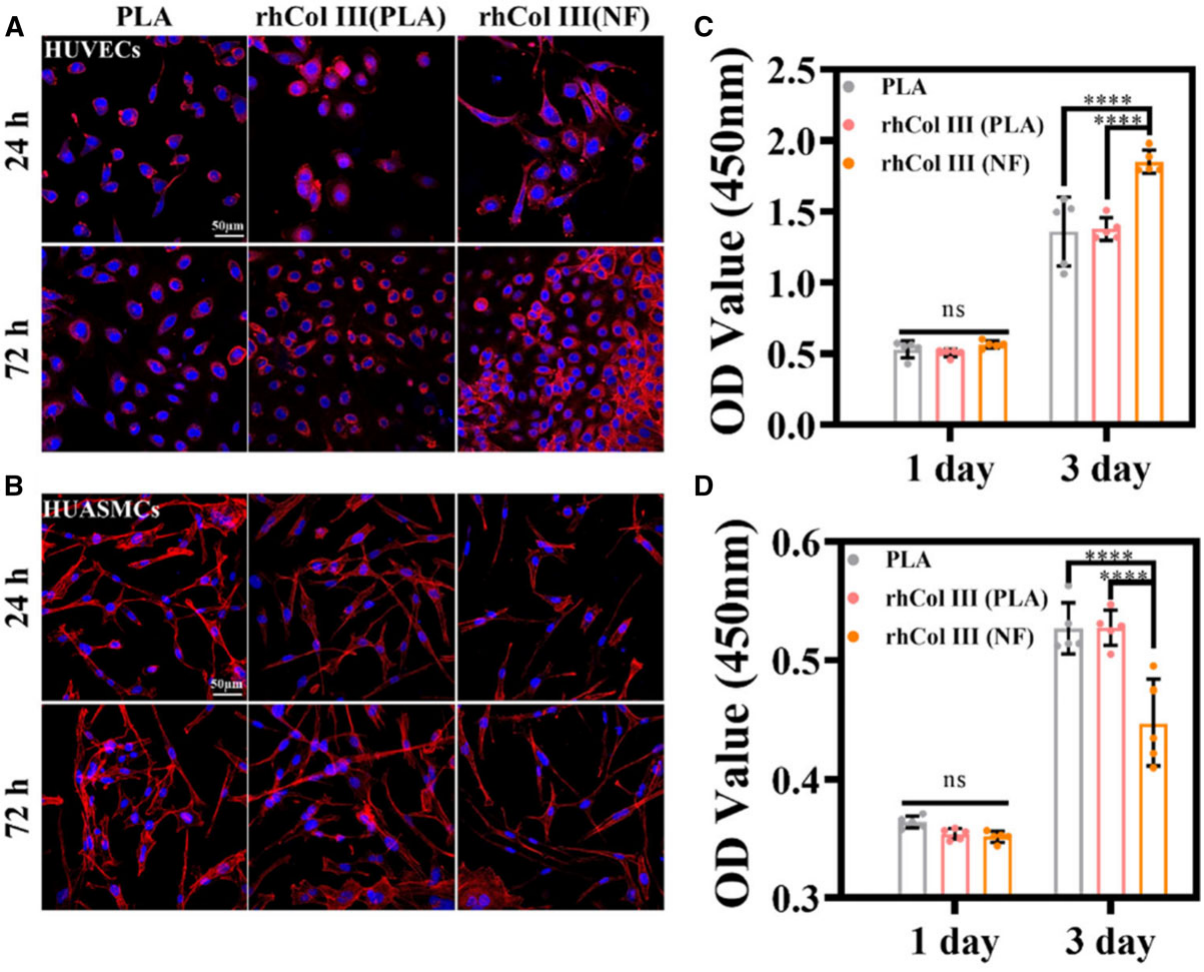
Cytotoxicity. (**A**) Immunofluorescent images of HUVECs stained by cyclopeptide (red) and DAPI (blue). (**B**) Cell viability of HUVECs cultured on the samples after 24 and 72 h. (**C**) Immunofluorescent images of HUASMCs stained by cyclopeptide (red) and DAPI (blue). (**D**) Cell viability of HUASMCs cultured on the samples after 24 and 72 h. *P < 0.05, **P < 0.01, ***P < 0.001, ****P < 0.0001 (one-way ANOVA, error bars are defined as SD).

Stent implantation destroys the endothelial layer of blood vessels, leading to inflammation, hyperplasia and thrombosis. Therefore, re-endothelialization on the inner surface of the stent is essential to reduce inflammation and promote blood compatibility after implantation. To evaluate the proliferation of ECs and SMCs on the rhCOL III (NF) coating, CCK-8 assay and TRITC-phalloidin staining were performed to monitor the cell viability and morphology. As shown in Fig. 4A and B, the proliferation of HUVECs had no significant difference among PLA, rhCOL III (PLA) and rhCOL III (NF) samples after 24 h. However, the cell morphology on the PLA was different. The stretching of HUVECs on the rhCOL III (NF) coating increased cell adhesiveness and spreading areas, which was beneficial to multiplication. The number of adhered HUVECs was significantly higher on the rhCOL III (NF) substrate than on the others after 72 h. The rhCOL III (NF) coating vastly enhanced the proliferation of HUVECs compared to rhCOL III (PLA), and most cells were spindle shaped, which indicated healthy cell morphology.

In contrast, the rhCOL III (NF) coating showed essential inhibition of SMC proliferation (Fig. 4D). The viability of SMC on the rhCOL III (PLA) was as high as that on the bare PLA substrate, which exhibited that rhCOL III had no inhibition or promotion of the proliferation of SMCs. The resistance of SMC proliferation on the rhCOL III (NF) coating might be attributed to the NF layer. The ECM structure mimetic NF layer provided a 3D nanofibrous substrate and supported cell/material interaction. As a result, the fibrous substrate increased the expression of contractile markers [40] and then tended to suppress SMC proliferation.

These results indicate that the rhCOL III (NF) coating fulfills the requirements of the intravascular stent for endothelial healing and inhibitory SMC proliferation.

### Inflammatory response of the coatings *in vivo* and *in vitro*

**Figure 5.**
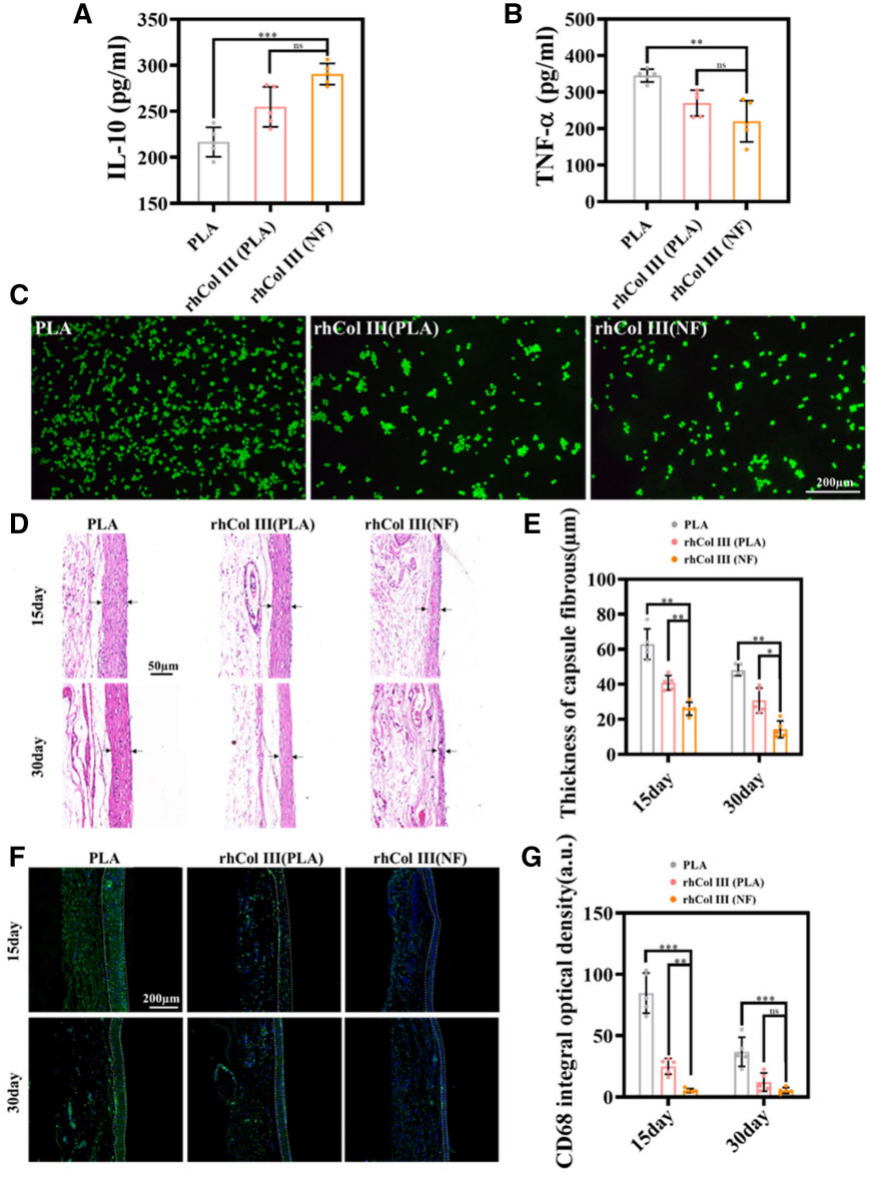
Anti-inflammatory capacity. (**A**) TNF-ɑ and (**B**) IL-10 levels expressed by RAW 264.7 cells, as detected by ELISA. (**C**) Fluorescent images of RAW 264.7 cells. (**D**) Photographs of H&E staining of the harvested tissues 15 and 30 days after subcutaneous implantation. (**E**) The thickness of the fibrous capsules of different samples on Days 15 and 30. (**F**) Photographs of CD68 staining (CD68 green and DAPI blue) of the harvested tissues 15 and 30 days after subcutaneous implantation. (**G**) The analysis of CD68 fluorescence signals of the capsule. *P < 0.05, **P < 0.01 and ***P < 0.001, ****P < 0.0001 (one-way ANOVA, error bars represent the SD).

Inflammatory cells adhere to medical devices after implantation, leading to inflammatory responses around the material/tissue interface. This causes morphology differentiation and the secretion of inflammatory cytokines [41, 42]. To evaluate the level of macrophage inflammation *in vitro*, RAW 264.7 mouse leukemia cells (RAW cells) were cultured on the PLA sheets, and the morphology and inflammatory cytokines were monitored. TNF-ɑ is a pro-inflammatory cytokine produced by activated macrophages [43], and IL-10 is an anti-inflammatory cytokine secreted by regulatory macrophages [44]. The expression of TNF-ɑ and IL-10 in the cell supernatant was detected by ELISA. According to the images of FDA-stained RAW cells, more spindle-shaped activated cells were observed on the bare PLA sheets, and the pseudopodia had elongated (Fig. 5E). The number of differentiated macrophages on the PLA substrate is higher than rhCOL III (PLA) and rhCOL III (NF). Comparatively, the rhCOL III (PLA) and rhCOL III (NF) groups inhibited the differentiation of RAW cells. The expression of TNF-ɑ was higher in the supernatant of the PLA group, while the expression of IL-10 was lower than that of rhCOL III (PLA) and rhCOL III (NF). Moreover, there was no significant difference between the expression of rhCOL III groups, which proved that rhCOL III effectively suppressed inflammatory responses.

For further evaluation of the anti-inflammation capacity, the PLA sheets with coatings were subcutaneously implanted in Sprague-Dawley rats. The sheets attract inflammatory cells and result in the formation of a fibrous capsule around the material. The thickness of the fibrous capsule is an essential indication of the inflammation level, and severe inflammation would bring about a thicker fibrous coating [45, 46]. Figure 5F shows the H&E-stained tissue sections 15 and 30 days after subcutaneous implantation. The capsule fibrous thickness of the bare PLA, rhCOL III (PLA), and rhCOL III (NF) groups was 62.947 ± 8.716 and 48.126 ± 3.190 µm, 40.879 ± 4.168 and 30.751 ± 7.108 µm and 26.092 ± 3.700 and 14.357 ± 4.781 µm after 15 and 30 days, respectively. The encapsulation of rhCOL III (NF) was significantly thinner than rhCOL III (PLA) and bare PLA sheets (Fig. 5F). At these times points, the dual ECM-mimetic coating exhibited good anti-inflammatory capacities and decreased the thickness of the fibrous capsule via increased immobilization of rhCOL III and fibrous structure.

Inflammatory cell infiltration can reveal the degree of inflammatory response. For further inflammatory evaluation, CD68 fluorescence staining was performed to observe macrophage distribution [47]. As shown in Fig. 5G, fewer fluorescent spots were observed in the newly formed fibrous capsules of rhCOL III (NF) sheets, compared with bare PLA and rhCOL III (PLA). This demonstrated much more inflammatory cells aggregated and infiltrated the capsules of PLA samples at 15 and 30 days. Moreover, there were few macrophages spotted on the rhCOL III (NF) substrates, which could indicate the material caused mild inflammation during implantation. As a result, the thickness and the macrophage distribution in the fibrous capsules showed a similar trend, in which dual ECM-mimetic coating resulted in lower inflammatory responses and better biocompatibility, compared with single ECM component mimetic coating.

### *In vivo* stent implantation experiment

The PLA vascular stent is a promising absorbable blood-contacting device. When the stent has been implanted for a long time, before complete degradation, chronic inflammation contributes to late stent restenosis and disordered endothelium layer [48]. To evaluate intimal hyperplasia and situ endothelialization *in vivo*, bare PLA and rhCOL III (NF)-coated stents were implanted in the rabbit abdominal aorta model and harvested after implantation for three months.

**Figure 6.**
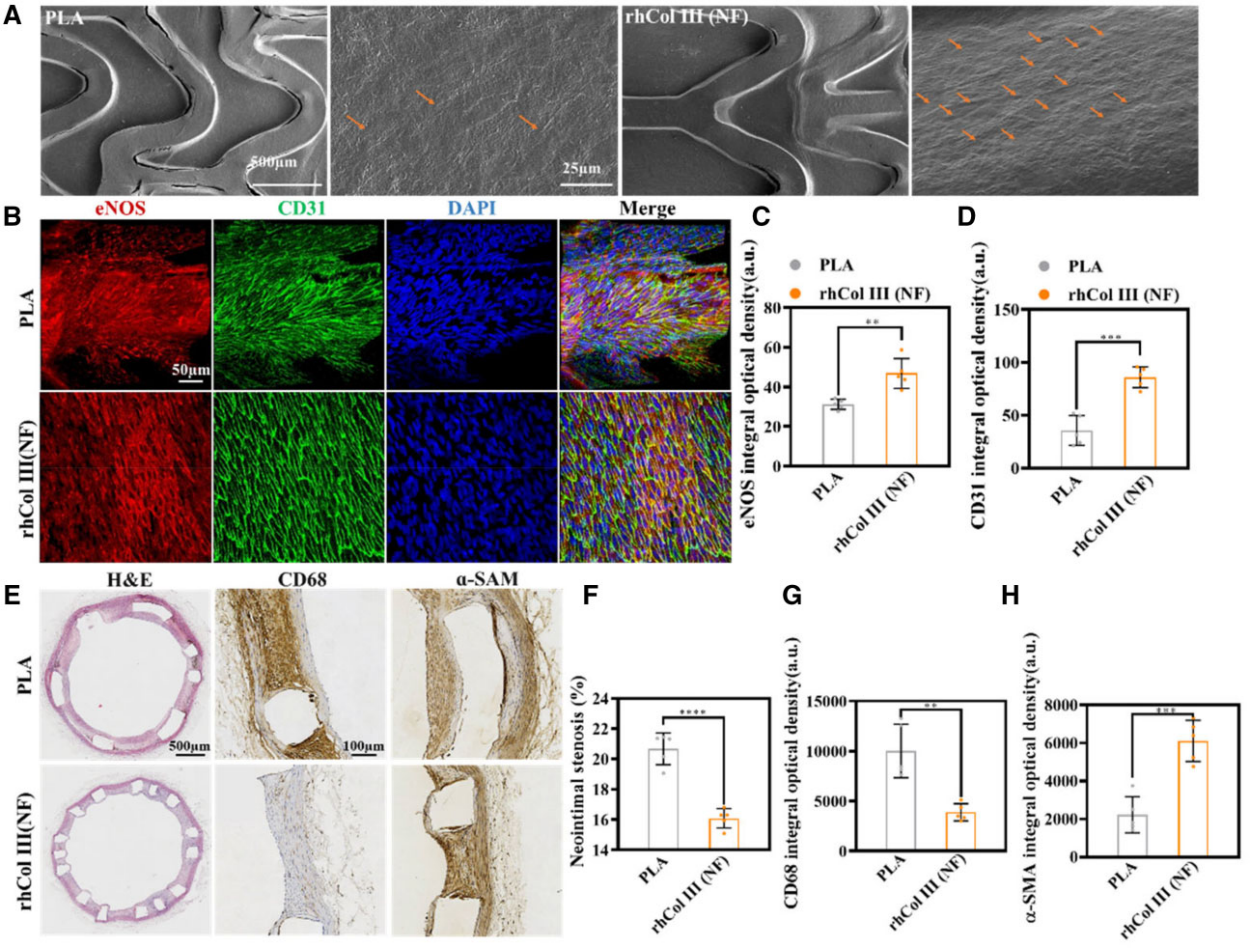
*In vivo* histocompatibility of the vascular stent. (**A**) Morphology of the vascular inner wall of the stent after implantation in the abdominal aorta of New Zealand rabbits, as observed by SEM. The cells are marked with orange arrows. (**B**) Immunofluorescence analysis of the neointima inside the vascular stents (eNOS red, CD31 green and DAPI blue). (**C**) Quantitative analysis of eNOS expression and (**D**) CD31 expression. (**E**) Histological and immunohistochemical analysis of the cross-section of the vascular structure, including stents. (**F**) Quantitative analysis of neointimal stenosis, (**G**) CD68 expression and (**H**) ɑ-SMA expression in the cross-section of the vascular structure, including stents. *P < 0.05, **P < 0.01 and ***P < 0.001, ****P < 0.0001 (*t*-test, error bars are defined as SD).

The cell morphology on the neointima of the inner surface of the lumen is shown in Fig. 6A. From the SEM result, there were few cells on the bare PLA stent after 3 months, and the growth of cells was disordered. The irregular state of the cells indicated different cell classes [26] and a low level of endothelialization. However, compared with the bare PLA stent, the number of cells increased, and the cells proliferated along the same direction on the rhCOL III (NF) coated stent. CD31 is a membrane receptor expressed by ECs to regulate the response to biomechanical stimuli [49] and was expressed around the EC edge. Besides, high CD31 expression indicated healthy and functioning ECs [13]. ECs secrete endothelial nitric oxide synthase (eNOS) to circulate nitric oxide metabolites and regulate blood pressure [50, 51]. The eNOS expression demonstrates the physiology of blood vessels and endothelialization degree [52]. For further assessment of the cell type, morphology, and distribution of the neointima, the inner surface of the lumen was stained by immunofluorescence (anti-CD31 and anti-eNOS) and observed by LSCM in Fig. 6B. The immunofluorescent image is in agreement with the SEM results. From the quantitative analysis in Fig. 6D, the CD31 expression of the inner cell layer of rhCOL III (NF) was higher than that of the bare PLA stent after 3 months. A similar trend was also observed in the eNOS expression, compared with CD31 expression in the tissue. The high expression of eNOS in the rhCOL III (NF) group indicated that the regenerated endothelium was healthy and intact. As shown in Fig. 6B, ECs did not cover the inner surface of the bare PLA stent completely, while the rhCOL III (NF) surface was covered by ECs arranged closely and in the same direction. Hence, the above result demonstrates the rhCOL III (NF) coating enhanced EC proliferation and accelerated the recovery of the endothelium layer.

For further evaluation, the stent cross-section with surrounding tissue was stained by H&E and immunohistochemistry antibodies (anti-CD68 and anti-ɑ-SMA) to investigate the restenosis rate, inflammation and endothelialization. Inflammatory responses could not be avoided after implantation and endothelial dysfunction is linked to inflammatory responses [53, 54]. The propped stent injured the endothelium cells of the blocked blood vessels and FBR was maintained until the complete degradation of the stent. Serious inflammatory responses lead to excessive thickening of vascular intima and restenosis. In Fig. 6E, numerous CD68-positive macrophages aggregated around the bare PLA stent struts after 3 months. Moreover, there were few macrophages in the neointima, and the activated macrophages mainly aggregated along the bare PLA struts, which indicated acute inflammation occurred and was sustained prior to collection from the New Zealand rabbits. In contrast, the rhCOL III (NF) coating suppressed inflammation remarkably after implantation, and few CD68-positive macrophages were observed around the coated struts, which suggests that the rhCOL III (NF) stent caused mild inflammatory responses.

Neointimal hyperplasia is a considerable threat to stent failure. At present, excessive proliferation of SMCs is considered the major contributor to the abnormal thickening of intimal and ISR [55, 56]. SMCs in a contractile phenotype have a low proliferative rate, but after vascular injury, SMCs turn to a synthetic phenotype and undergo a proliferative response [57–59]. The switch of the SMC phenotype from contractile to proliferative leads to the deterioration of blood vessels and excessive hyperplasia. The ɑ-SMA was specifically expressed in SMCs with contractile phenotype [26]. The expression of ɑ-SMA around the rhCOL III (NF) stent was much higher than that of the bare PLA stent (Fig. 6H), indicating that most SMCs had low proliferative capacity and the neointima of rhCOL III (NF) groups was more mature. The maintenance of the contractile phenotype might arise from mild inflammatory responses, and a low level of inflammation would not stimulate SMCs and could induce the contractile phenotype of SMC. As a result, decreasing the acute proliferation of SMCs contributed thicker neointima and less lumen loss.

As shown in Fig. 6E, the restenosis rate of the bare PLA stent was as high as 20.68 ± 1.05%, and that of the rhCOL III (NF) coating was about 16.10 ± 0.64% after 3 months of implantation. The rhCOL III (NF) coating inhibited intimal hyperplasia and reduced restenosis by suppressing the aggregation of inflammatory cells and excessive proliferation of SMCs. Inflammation and coagulation were closely linked, due to the damage of EC function after implantation, and then persistent inflammation gave rise to abnormal hyperplasia and delayed re-endothelialization. The fibrous ad-layer and rhCOL III mimicked ECM dual structure and component to enable the injured blood vessel in a low level of inflammation, and endothelialization accelerated without excessive proliferation of SMCs.

From the *in vivo* results, rhCOL III (NF) is an ideal vascular stent coating, benefiting from the reduction of restenosis on account of a mild inflammation response, contractile SMC induction, and endothelialization acceleration.

## Conclusion

In this work, an ECM-mimetic coating with dual bionics was prepared successfully on PLA stents, where the biomimetics were driven by structure mimicry and component/function mimicry. The NFs on the PLA constructed an ECM fibrous structure as well as a three-dimensional reservoir to support the amplified immobilization of rhCol III. Then, rhCol III, a multi-functional collagen, provided reliable anti-coagulation and anti-inflammatory properties and promoted EC proliferation. As a result, this ECM-mimetic rhCOL III (NF) coating possessed superior anti-thrombosis and anti-inflammatory capacities, promoted re-endothelialization and inhibited SMC proliferation by inducing contractile SMC phenotype *in vivo*. In view of the stable interface and multiple functions endowed by the fibrous ad-layer and tailored rhCOL III, the rhCOL III (NF) coating is a promising material for the efficient modification of cardiovascular stents.

## Supplementary Material

rbad055_Supplementary_Data

## References

[1] Sabatine MS , Bergmark BA, Murphy SA, O'Gara PT, Smith PK, Serruys PW, Kappetein AP, Park SJ, Park DW, Christiansen EH, Holm NR, Nielsen PH, Stone GW, Sabik JF, Braunwald E. Percutaneous coronary intervention with drug-eluting stents versus coronary artery bypass grafting in left main coronary artery disease: an individual patient data meta-analysis. Lancet 2021;398:2247–57. 34793745 10.1016/S0140-6736(21)02334-5

[2] Wang YB , Li GC, Yang L, Luo RF, Guo GY. Development of innovative biomaterials and devices for the treatment of cardiovascular diseases. Adv Mater. 2022;34:2201971. 10.1002/adma.202201971 35654586

[3] Le Bras A. Drug-eluting stents versus bare-metal stents for vein-graft PCI. Nat Rev Cardiol 2018;15:442. 10.1038/s41569-018-0042-8 29867189

[4] Bangalore S , Toklu B, Amoroso N, Fusaro M, Kumar S, Hannan EL, Faxon DP, Feit F. Bare metal stents, durable polymer drug eluting stents, and biodegradable polymer drug eluting stents for coronary artery disease: mixed treatment comparison meta-analysis. BMJ 2013;347:f6625. 24212107 10.1136/bmj.f6625 PMC3898413

[5] Farb A , Heller PF, Shroff S, Cheng L, Kolodgie FD, Carter AJ, Scott DS, Froehlich J, Virmani R. Pathological analysis of local delivery of paclitaxel via a polymer-coated stent. Circulation 2001;104:473–9. 11468212 10.1161/hc3001.092037

[6] Jaffe R , Strauss BH. Late and very late thrombosis of drug-eluting stents - Evolving concepts and perspectives. J Am Coll Cardiol 2007;50:119–27. 17616295 10.1016/j.jacc.2007.04.031

[7] Kleiner LW , Wright JC, Wang Y. Evolution of implantable and insertable drug delivery systems. J Control Release 2014;181:1–10. 24548479 10.1016/j.jconrel.2014.02.006

[8] Wang Y , Zhang X. Vascular restoration therapy and bioresorbable vascular scaffold. Regen Biomater 2014;1:49–55. 26816624 10.1093/rb/rbu005 PMC4669005

[9] Zhang H , Zhang W, Qiu H, Zhang G, Li X, Qi H, Guo J, Qian J, Shi X, Gao X, Shi D, Zhang D, Gao R, Ding J. A biodegradable metal-polymer composite stent safe and effective on physiological and serum-containing biomimetic conditions. Adv Healthcare Mater 2022;11:2201740. 10.1002/adhm.202201740 36057108

[10] Cao D , Ding J. Recent advances in regenerative biomaterials. Regen Biomater 2022;9. 10.1093/rb/rbac098. PMC9745784 36518879

[11] Buchanan K , Gajanana D, Iantorno M, Torguson R, Rogers T, Ben-Dor I, Satler L, Waksman R. Drug-eluting stent therapy for recurrent in-stent restenosis following failed vascular brachytherapy. J Am Coll Cardiol 2018;72:B185–B186.

[12] Mauri L , Silbaugh TS, Garg P, Wolf RE, Zelevinsky K, Lovett A, Varma MR, Zhou Z, Normand S-LT. Drug-eluting or bare-metal stents for acute myocardial infarction. N Engl J Med 2008;359:1330–42. 18815397 10.1056/NEJMoa0801485

[13] Wang YN , Wu HS, Zhou ZY, Maitz MF, Liu KP, Zhang B, Yang L, Luo RF, Wang YB. A thrombin-triggered self-regulating anticoagulant strategy combined with anti-inflammatory capacity for blood-contacting implants. Sci Adv 2022;8:eabm3378. 10.1126/sciadv.abm3378 PMC8896797 35245113

[14] Wang J , Xue YF, Liu J, Hu M, Zhang H, Ren KF, Wang YB, Ji J. Hierarchical capillary coating to biofunctionlize drug-Eluting stent for improving endothelium regeneration. Research 2020;2020:1458090. doi: 10.34133/2020/1458090. 32885169 PMC7455884

[15] Zhou ZY , Luo RF, Chen L, Hu C, Chen C, Maitz MF, Li LH, Yang L, Deng D, An YQ, Wu HS, Yang Y, Dai Y, Xin JY, Wang YB. Dressing blood-contacting devices with platelet membrane enables large-scale multifunctional biointerfacing. Matter 2022;5:2334–51.

[16] Chen L , Zhou Z, Hu C, Maitz MF, Yang L, Luo R, Wang Y. Platelet membrane-coated nanocarriers targeting plaques to deliver anti-CD47 antibody for atherosclerotic therapy. Research 2022;2022:9845459. doi: 10.34133/2022/9845459. PMC8791388 35118420

[17] Qiu H , Tu Q, Gao P, Li X, Maitz MF, Xiong K, Huang N, Yang Z. Phenolic-amine chemistry mediated synergistic modification with polyphenols and thrombin inhibitor for combating the thrombosis and inflammation of cardiovascular stents. Biomaterials 2021;269:120626. 33418199 10.1016/j.biomaterials.2020.120626

[18] Yang J , Zeng Y, Zhang C, Chen YX, Yang ZY, Li YJ, Leng XG, Kong DL, Wei XQ, Sun HF, Song CX. The prevention of restenosis in vivo with a VEGF gene and paclitaxel co-eluting stent. Biomaterials 2013;34:1635–43. 23199742 10.1016/j.biomaterials.2012.11.006

[19] Zhang B , Qin YM, Yang L, Wu Y, Chen NY, Li MY, Li YY, Wan HN, Fu DH, Luo RF, Yuan L, Wang YB. A polyphenol-network-mediated coating modulates inflammation and vascular healing on vascular stents. ACS Nano 2022;16:6585–97. 35301848 10.1021/acsnano.2c00642

[20] Zhang B , Yao RJ, Maitz MF, Mao GW, Hou Z, Yu HC, Luo RF, Wang YB. Poly (dimethyl diallyl ammonium chloride) incorporated multilayer coating on biodegradable AZ31 magnesium alloy with enhanced resistance to chloride corrosion and promoted endothelialization. Chem Eng J 2021;421:127724.

[21] Wu HS , He Q, Li L, Li LH, Zhou ZY, Chen NY, Yang M, Luo QF, Zhang B, Luo RF, Yang L, Wang YB. A facile and versatile superhydrophilic coating on biodegradable PLA stent with stepwise assembly of metal/phenolic networks for mimicking endothelium function. Chem Eng J 2022;427:130932.

[22] Ge Y , Guo G, Liu K, Yang F, Yang L, Wang Y, Zhang X. A strategy of functional crosslinking acellular matrix in blood-contacting implantable devices with recombinant humanized collagen type III (rhCOLIII). Compos B: Eng 2022;234:109667.

[23] Liu W , Merrett K, Griffith M, Fagerholm P, Dravida S, Heyne B, Scaiano JC, Watsky MA, Shinozaki N, Lagali N, Munger R, Li F. Recombinant human collagen for tissue engineered corneal substitutes. Biomaterials 2008;29:1147–58. 18076983 10.1016/j.biomaterials.2007.11.011

[24] Hu C , Liu W, Long L, Wang Z, Zhang W, He S, Lu L, Fan H, Yang L, Wang Y. Regeneration of infarcted hearts by myocardial infarction-responsive injectable hydrogels with combined anti-apoptosis, anti-inflammatory and pro-angiogenesis properties. Biomaterials 2022;290:121849– 36252427 10.1016/j.biomaterials.2022.121849

[25] Hua C , Zhu Y, Xu W, Ye S, Zhang RG, Lu L, Jiang SB. Characterization by high-resolution crystal structure analysis of a triple-helix region of human collagen type III with potent cell adhesion activity. Biochem Biophys Res Commun 2019;508:1018–23. 30545625 10.1016/j.bbrc.2018.12.018 PMC7092849

[26] Yang L , Wu H, Lu L, He Q, Xi B, Yu H, Luo R, Wang Y, Zhang X. A tailored extracellular matrix (ECM)-mimetic coating for cardiovascular stents by stepwise assembly of hyaluronic acid and recombinant human type III collagen. Biomaterials 2021;276:121055. 34371447 10.1016/j.biomaterials.2021.121055

[27] Ravichandran R , Astrand C, Patra HK, Turner APF, Chotteau V, Phopase J. Intelligent ECM mimetic injectable scaffolds based on functional collagen building blocks for tissue engineering and biomedical applications. RSC Adv 2017;7:21068–78.

[28] Salber J , Grater S, Harwardt M, Hofmann M, Klee D, Dujic J, Huang JH, Ding JD, Kippenberger S, Bernd A, Groll J, Spatz JP, Moller M. Influence of different ECM mimetic peptide sequences embedded in a nonfouling environment on the specific adhesion of human-skin keratinocytes and fibroblasts on deformable substrates. Small 2007;3:1023–31. 17455182 10.1002/smll.200600596

[29] Sousa MP , Caridade SG, Mano JF. Control of cell alignment and morphology by redesigning ECM-Mimetic nanotopography on multilayer membranes. Adv Healthcare Mater 2017;6:1601462. 10.1002/adhm.201601462 PMC6398568 28371516

[30] Natsume K , Nakamura J, Sato K, Ohtsuki C, Sugawara-Narutaki A. Biological properties of self-assembled nanofibers of elastin-like block polypeptides for tissue-engineered vascular grafts: platelet inhibition, endothelial cell activation and smooth muscle cell maintenance. Regen Biomater 2023;10:rbac111. 36683748 10.1093/rb/rbac111 PMC9845521

[31] Geyer F , D'Acunzi M, Yang CY, Muller M, Baumli P, Kaltbeitzel A, Mailander V, Encinas N, Vollmer D, Butt HJ. How to coat the inside of narrow and long tubes with a super-liquid-repellent layer—a promising candidate for antibacterial catheters. Adv Mater 2019;31:1801324. 10.1002/adma.201801324 30417451

[32] Li MY , Liu KP, Liu WQ, Chen NY, Wang YA, Zhang FJ, Luo QF, Yang L, Luo RF, Wang YB. A universal anti-thrombotic and antibacterial coating: a chemical approach directed by Fenton reaction and silane coupling. Appl Surf Sci 2022;600:154143.

[33] Park KS , Kang SN, Kim DH, Kim HB, Im KS, Park W, Hong YJ, Han DK, Joung YK. Late endothelial progenitor cell-capture stents with CD146 antibody and nanostructure reduce in-stent restenosis and thrombosis. Acta Biomater 2020;111:91–101. 32434081 10.1016/j.actbio.2020.05.011

[34] Yang Y , Gao P, Wang J, Tu QF, Bai L, Xiong KQ, Qiu H, Zhao X, Maitz MF, Wang HY, Li XY, Zhao Q, Xiao Y, Huang N, Yang ZL. Endothelium-mimicking multifunctional coating modified cardiovascular stents via a stepwise metal-catechol-(amine) surface engineering strategy. Research 2020;2020:9203906. doi: 10.34133/2020/9203906. PMC7196174 32405627

[35] Li B , Jing H, Sun Z, Wang X, Kong D, Liu J, Leng X, Wang Z. Comprehensive analyses and prioritization of various swim bladder-derived extracellular matrix in the application of heart valve prosthesis. Smart Mater Med 2021;2:209–18.

[36] Li J , Wang S, Sheng Y, Liu C, Xue Z, Tong P, Guan S. Designing HA/PEI nanoparticle composite coating on biodegradable Mg-Zn-Y-Nd alloy to direct cardiovascular cells fate. Smart Mater Med 2021;2:124–33.

[37] Shen Y , Zhang W, Xie Y, Li A, Wang X, Chen X, Liu Q, Wang Q, Zhang G, Liu Q, Liu J, Zhang D, Zhang Z, Ding J. Surface modification to enhance cell migration on biomaterials and its combination with 3D structural design of occluders to improve interventional treatment of heart diseases. Biomaterials 2021;279:121208. 34749074 10.1016/j.biomaterials.2021.121208

[38] Si YF , Dong ZC, Jiang L. Bioinspired designs of superhydrophobic and superhydrophilic materials. ACS Central Sci 2018;4:1102–12. 10.1021/acscentsci.8b00504 PMC6161061 30276243

[39] Waller AP , Wolfgang KJ, Kerlin BA. The hypofibrinolytic defect of nephrotic syndrome is directly proportional to fibrin network density. Blood 2018;132:1218.

[40] Woods I , Black A, Jockenhoevel S, Flanagan TC. Harnessing topographical & biochemical cues to enhance elastogenesis by paediatric cells for cardiovascular tissue engineering applications. Biochem Biophys Res Commun 2019;512:156–62. 30878185 10.1016/j.bbrc.2019.03.026

[41] Denburg JA. Microenvironmental influences on inflammatory cell-differentiation. Allergy 1995;50:25–8. 10.1111/j.1398-9995.1995.tb04272.x 7677230

[42] Sousa AB , Aguas AP, Barbosa MA, Barbosa JN. Immunomodulatory biomaterial-based wound dressings advance the healing of chronic wounds via regulating macrophage behavior. Regen Biomater 2022;9;rbac065. 10.1093/rb/rbac065. PMC9566965 36267154

[43] Tiegs G , Horst AK. TNF in the liver: targeting a Central player in inflammation. Semin Immunopathol 2022;44:445–59. 35122118 10.1007/s00281-022-00910-2 PMC9256556

[44] Smallie T , Ricchetti G, Horwood NJ, Feldmann M, Clark AR, Williams LM. IL-10 inhibits transcription elongation of the human TNF gene in primary macrophages. J Exp Med 2010;207:2081–8. 20805562 10.1084/jem.20100414 PMC2947066

[45] Amer LD , Saleh LS, Walker C, Thomas S, Janssen WJ, Alper S, Bryant SJ. Inflammation via myeloid differentiation primary response gene 88 signaling mediates the fibrotic response to implantable synthetic poly(ethylene glycol) hydrogels. Acta Biomater 2019;100:105–17. 31568879 10.1016/j.actbio.2019.09.043 PMC6980661

[46] Lu J , Zhuang WH, Li LH, Zhang B, Yang L, Liu DP, Yu HC, Luo RF, Wang YB. Micelle-Embedded layer-by-Layer coating with catechol and phenylboronic acid for tunable drug loading, sustained release, mild tissue response, and selective cell fate for re-endothelialization. ACS Appl Mater Interfaces 2019;11:10337–50. 30753784 10.1021/acsami.9b01253

[47] Lv LL , Tang PMK, Li CJ, You YK, Li JH, Huang XR, Ni J, Feng M, Liu BC, Lan HY. The pattern recognition receptor, mincle, is essential for maintaining the M1 macrophage phenotype in acute renal inflammation. Kidney Int 2017;91:587–602. 28017324 10.1016/j.kint.2016.10.020

[48] Danzi GB , Piccolo R. Bioresorbable scaffolds versus metallic stents in routine PCI. N Engl J Med 2017;377:1790– 10.1056/NEJMc1711903 29094853

[49] Caligiuri G. Mechanotransduction, immunoregulation, and metabolic functions of CD31 in cardiovascular pathophysiology. Cardiovasc Res 2019;115:1425–34. 31119265 10.1093/cvr/cvz132

[50] Leo F , Suvorava T, Heuser SK, Li JJ, LoBue A, Barbarino F, Piragine E, Schneckmann R, Hutzler B, Good ME, Fernandez BO, Vornholz L, Rogers S, Doctor A, Grandoch M, Stegbauer J, Weitzberg E, Feelisch M, Lundberg JO, Isakson BE, Kelm M, Cortese-Krott MM. Red blood cell and endothelial eNOS independently regulate circulating nitric oxide metabolites and blood pressure. Circulation 2021;144:870–89. 34229449 10.1161/CIRCULATIONAHA.120.049606 PMC8529898

[51] Venugopal SK , Devaraj S, Yuhanna I, Shaul P, Jialal I. Demonstration that C-reactive protein decreases eNOS expression and bioactivity in human aortic endothelial cells. Circulation 2002;106:1439–41. 12234944 10.1161/01.cir.0000033116.22237.f9

[52] Dou J , Yang R, Jin X, Li P, Han X, Wang L, Chi B, Shen J, Yuan J. Nitric oxide-releasing polyurethane/S-nitrosated keratin mats for accelerating wound healing. Regen Biomater 2022;9;rbac006. doi: 10.1093/rb/rbac006. PMC9113238 35592138

[53] Pober JS , Sessa WC. Evolving functions of endothelial cells in inflammation. Nat Rev Immunol 2007;7:803–15. 17893694 10.1038/nri2171

[54] Kanter JE. FOXP1: a gatekeeper of endothelial cell inflammation. Circ Res 2019;125:606–8. 31465266 10.1161/CIRCRESAHA.119.315687 PMC6750721

[55] Kearney M , Pieczek A, Haley L, Losordo DW, Andres V, Schainfeld R, Rosenfield K, Isner JM. Histopathology of in-stent restenosis in patients with peripheral artery disease. Circulation 1997;95:1998–2002. 9133506 10.1161/01.cir.95.8.1998

[56] Deuse T , Hua XQ, Wang D, Maegdefessel L, Heeren J, Scheja L, Bolanos JP, Rakovic A, Spin JM, Stubbendorff M, Ikeno F, Langer F, Zeller T, Schulte-Uentrop L, Stoehr A, Itagaki R, Haddad F, Eschenhagen T, Blankenberg S, Kiefmann R, Reichenspurner H, Velden J, Klein C, Yeung A, Robbins RC, Tsao PS, Schrepfer S. Dichloroacetate prevents restenosis in preclinical animal models of vessel injury. Nature 2014;509:641–4. 24747400 10.1038/nature13232 PMC4323184

[57] Boettger T , Beetz N, Kostin S, Schneider J, Kruger M, Hein L, Braun T. Acquisition of the contractile phenotype by murine arterial smooth muscle cells depends on the Mir143/145 gene cluster. J Clin Investig 2009;119:2634–47. 19690389 10.1172/JCI38864 PMC2735940

[58] Qiu H , Qi PK, Liu JX, Yang Y, Tan X, Xiao Y, Maitz MF, Huang N, Yang ZL. Biomimetic engineering endothelium-like coating on cardiovascular stent through heparin and nitric oxide-generating compound synergistic modification strategy. Biomaterials 2019;207:10–22. 30947118 10.1016/j.biomaterials.2019.03.033

[59] Jeong K , Murphy JM, Kim J-H, Campbell PM, Park H, Rodriguez Y, Choi C, Kim J-S, Park S, Kim HJ, Scammell JG, Weber DS, Honkanen RE, Schlaepfer DD, Ahn E-YE, Lim S-TS. FAK activation promotes SMC dedifferentiation via increased DNA methylation in contractile genes. Circ Res 2021;129:E215–E233. 34702049 10.1161/CIRCRESAHA.121.319066 PMC8639767

