# Supplementary material for: An extracellular matrix-mimetic coating with dual bionics for cardiovascular stents
Source: Regen Biomater. 2023 May 30;10:rbad055. doi: 10.1093/rb/rbad055 (PMC10287914; doi:10.1093/rb/rbad055)
Supplement: rbad055_Supplementary_Data [file rbad055_supplementary_data.docx]

**An extracellular matrix (ECM)-mimetic coating with dual bionics for Cardiovascular Stents**

Nuoya Chen^a^, Mingyu Li^a^, Haoshaung Wu^a^, Yumei Qin^a^, Jian Wang^b^, Kai Xu^c^, Rifang Luo^a^, Li Yang^a,^ , Yunbing Wang^a,^ *, Xingdong Zhang^a^

^a.^National Engineering Research Center for Biomaterials, Sichuan University, Chengdu, 610064, China

^b.^Shanxi Key Laboratory of Functional Proteins, Shanxi Jinbo Bio-Pharmaceutical Co., Ltd., Taiyuan 030032, Shanxi, China

^c.^Department of Cardiology, General Hospital of Northern Theater Command, Shenyang, 110000, China

*: Corresponding author

E-mail address: yunbing.wang@scu.edu.cn (W. Yunbing)

**Supplementary information**

1. **Information of rhCOLIII**

The rhCOLIII was composed of 16 tandem repeats of the triple-helix fragment of T16WTp. This protein was expressed in Escherichia coli (E. coli) and purified to be endotoxin-free under GMP condition (Shanxi Jinbo Biomedicine Co., Ltd., China) [1].

**Table 1** **Amino acid sequences of the peptides.**

| ID | Sequence | Purity |
| --- | --- | --- |
| T16WTp | Ac-GERGAPGFRGPAGPNGIPGEKGPAGERGAP-NH2 | >95 % |

1. **Result and discussion**
   1. Water contact angles (WCA) of coatings

The superhydrophilic surface was observed on the rhCOL III(NF) in Fig. S1. The water contact angles (WCA) of PLA, NF, rhCOL III(PLA) and rhCOL III(NF) substrate were respectively about 89.8 º , 162.6 º, 54.4 º and less than 5 º. The rhCOL III(NF) coating was a superhydrophilic material containing single active constituent rhCOL III.


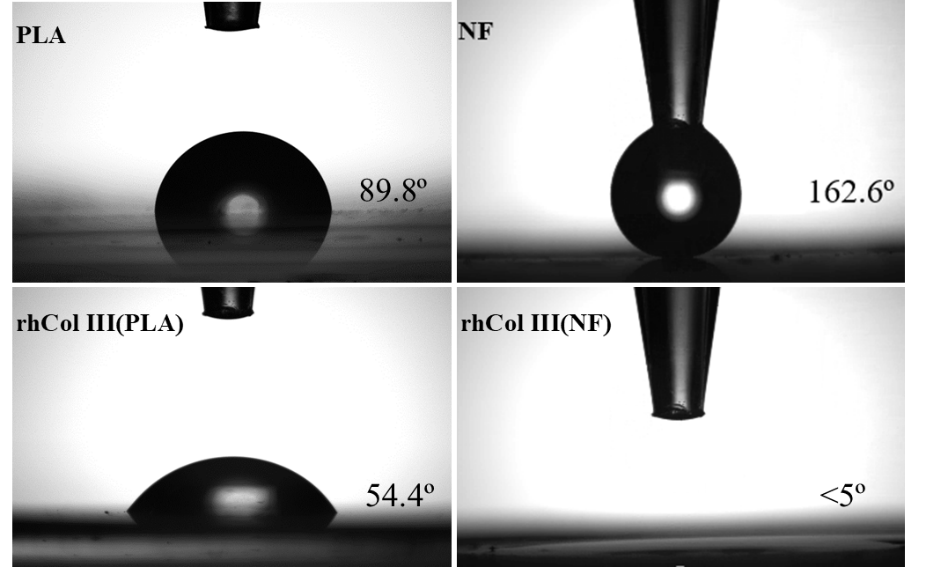


**Figure S1. The WCA of PLA, NF,** **rhCOL III(PLA) and rhCOL III(NF).**

- 1. The morphology of rhCOL III(NF) coating in the tube

The coating construction in the tube was different from that on the PLA substrate and the compact nanoparticles constituted the surface instead of nanaofilament.


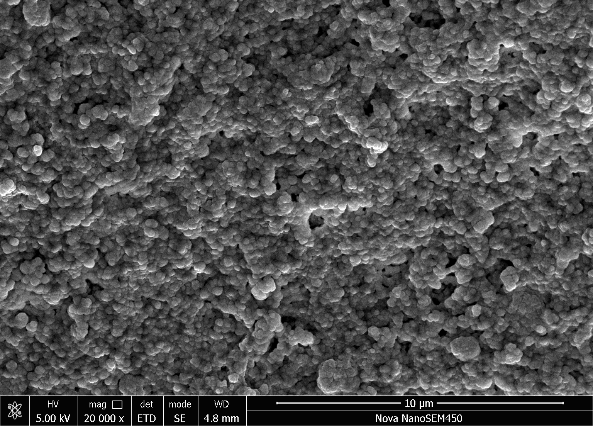


**Figure S2. The morphology of rhCOL III(NF) coating.**

**Reference**

[1] L. Yang, H. Wu, L. Lu, Q. He, B. Xi, H. Yu, R. Luo, Y. Wang, X. Zhang, A tailored extracellular matrix (ECM)-Mimetic coating for cardiovascular stents by stepwise assembly of hyaluronic acid and recombinant human type III collagen, Biomaterials 276 (2021). <https://doi.org/10.1016/j.biomaterials.2021.121055>.
